# Horse-related trauma in children and adults during a two year period

**DOI:** 10.1186/s13049-014-0040-8

**Published:** 2014-07-17

**Authors:** Jakob Altgärde, Stefan Redéen, Niclas Hilding, Peder Drott

**Affiliations:** 1 Department of Clinical and Experimental Medicine, Faculty of Health Sciences, University Hospital, Linköping University, Linköping, 581 85, Sweden; 2 Emergency Department, County Council of Östergötland, Linköping, 581 85, Sweden

**Keywords:** Accidents, Adults, Children, Costs, Fractures, Health care consumption, Horse, Trauma

## Abstract

**Introduction:**

Horse riding, with almost 200,000 participants, is the eighth most popular sport in Sweden. Severe injuries can occur with horse riding accidents which is well documented. This study was undertaken to investigate if injuries associated with horse riding are common, which type of injuries occur, what mechanisms are involved and to estimate the costs to the society.

**Material and methods:**

All patients attending the emergency department at Linköping University Hospital, during the years 2003-2004, due to horse related trauma were prospectively recorded. The patients were divided into two groups according to age, 147 children and 141 adults. The medical records were retrospectively scrutinized.

**Results:**

The most common mechanism of injury was falling from the horse. Most commonly, minor sprains and soft tissue injuries were seen, but also minor head injuries and fractures, mainly located in the upper limb. In total 26 adults and 37 children were admitted. Of these 63 patients 19 were considered having a serious injury. In total, four patients needed treatment in intensive care units.

The total cost in each group was 200,000 Euro/year.

**Conclusion:**

Horse riding is a sport with well known risks. Our results corresponds to the literature, however we have not observed the same incidence of serious injuries. In contrast we find these to be fairly uncommon. The injuries are mainly minor, with a small risk of long term morbidity. Over time regulations and safety equipment seem to have decreased the number of serious accidents.

## Introduction

With almost 200 000 participants in Sweden (2% of the Swedish population), horse riding is the eighth most popular sport [[1]]. The number of horses in Sweden has increased from 70,000 in 1974 to 270 000 in 2004 [[2]].

The number of horses in the county of Östergötland in 2004 was 14 356, this is the fourth highest number among the counties in Sweden [[2]]. Linköping community hosts one third of the members of The Swedish Equestrian Federation in the county of Östergötland.

The University Hospital in Linköping was chosen because it is the only hospital in the Linköping community and handles all emergency cases.

The Swedish Equestrian Federation reports that the interest for horse riding has increased over the years. The traditional use of horses as occupational animals has decreased, nowadays horse riding is mainly for recreation and competition.

The benefits from horse riding include learning to take responsibility and in calculating a greater respect for animals, however there are risks of injuries [[3]]. Former studies have postulated that the risk of injury during horse riding is at the same level as for most other sports performed by children (e.g. soccer, ice hockey). However, the severities of the injuries are disproportionately greater [[4]]. A study with focus on severity of injuries in childhood reviled that horse riding was the second worst mechanism of injury [[5]].

Results from previous studies shows that the most common horse related injuries are head injuries and fractures of the long bones. Horse related trauma is also more common among women, with a peak incidence at the age of 14 [[6]]. Horse-related trauma is expensive for the society and the affected person [[7]].

The aims of this study were to investigate if injuries associated with horse riding are as common as previously described, to determine the common types of injuries, and the mechanisms involved, and to estimate the costs to society.

## Material and methods

All patients attending the emergency department at the Linköping University Hospital during the years 2003-2004, due to accidents related to horse riding or horse handling, were prospectively recorded. The patients were divided into two groups according to age. One group under the age of 19 years (Children) and one group 19 years of age or older (Adults). There were 157 Children and 162 Adults. The medical records were retrospectively scrutinized for details about the accident, the severity of the injuries and the given medical treatment. Ten Children were excluded due to lack of information. Among the Adults, a total of 21 were excluded, 19 due to lack of information and two as they were participants in a professional racing competition. Remaining were 147 Children and 141 Adults.

We defined serious injuries as hospitalization for ≥3 days and/or sick leave for ≥1 month. No specific injury classifications were used.

Almost all injuries occurred during horse riding only a few during the care of horses.

An estimation of the number of horses in the Linköping community was achieved from the Swedish Equestrian Federation in the county of Östergötland. The city of Linköping hosts one third of the members of the Swedish Equestrian Federation in the county. We compared the ratio of members to the total numbers of horses in the county and estimated that there are approximately 4500 horses in Linköping community. This was compared with, and found adequate, with local registers in the community.

To estimate the incidence of horse related injuries we divided the mean frequency of accidents per day with the estimated total horse handling time per day from the questionnaire.

The hospital economists gave the current costs for included investigations and admissions.

All data were unidentified before analysis. The study was performed in accordance with the updated (2002) Helsinki Declaration and was approved by the Ethics Committee at the University Hospital in Linköping.

## Results

### Demographics

In total (Children/Adults) 147/141 were included. There were 144/128 girls/women and 3/13 boys/men in the study. Age ranged from 3.5 - 18.7/19.2-70.1 median age was 13/33. The majority 131/97 of accidents happened with the rider mounted on the horse and the remainder 16/44 whilst taking care of, feeding or loading the horse onto a trailer.

### Mechanism of injury

Several different mechanisms of injury were recognized. The most common mechanism of injury was falling off the horse 125/92. Of these 20/14 had additional mechanisms of injury to the fall. These mechanisms were: horse fell on 8/6, stepped on 6/3, kicked 3/2 and 3/3 riders got their foot stuck in a stirrup and 1/4 of these were dragged along by the horse. Other riders were solely kicked 11/23, stepped on 6/14, bitten 1/0, or pushed by the horse 1/0. Another 1/0 rider was injured when the horse suddenly stopped in front of an obstacle and 2/1 were squeezed between the horse and a wall. Additional 0/3 riders got a finger caught in the reins and were pulled along by the horse. One Adult hit an obstacle while riding. Five Adult were hit by the horse’s neck or head. In 1/2 the horse fell over the rider.

### Injuries

The majority of injuries were minor sprains and light soft tissue injuries. Some Children had potential serious injuries (Table 1).

**Table 1:** Types and numbers of injuries among children and adults in total

**N = Children/Adult**	**Contusions**	**Fractures**	**Distortions/Dislocations**	**Minor sprains and light soft tissue injuries**	**Minor head injuries**	**Splenic injury/traumatic liver rupture**	**Superficial injuries**	**N**
**Location of injury**								
N	6/9	33/42	4/16	93/68	33/11	1/1	7/19	177/169
Trunk	3/8	4/7	1/0	16/11		1/1	0/4	25/34
Back	0/0	2/3	0/1	14/11				16/15
Lower extremity	1/1	1/9	0/4	29/23			2/4	33/41
Upper extremity	1/0	22/18	1/11	24/20			1/2	49/51
Head and neck	1/0	4/5	2/0	10/3	33/11		4/9	54/28

In addition, three adults had hemo-/pneumothorax.

### Health care consumption

On presentation to the emergency department, 82/85 patients (56/60%) were isolated orthopaedic cases, 1/1 neurosurgical, 1/0 oro-maxillofacial, 0/1 hand surgeon, 0/1 Ear Nose and Throat surgeon and the remainder general surgical cases. Patients with multiple injuries were treated by the trauma team.

In total 37/26 were admitted, of which 4/9 patients were admitted for at least three days. The length of admission ranged from 1-14/1-7 days. Of these, 34/25 patients were admitted to the wards and 3/1 to the intensive care unit (ICU) (Tables 2 and 3).

**Table 2:** Type of injuries, treatment and time at ward in the children group

**Injury**	**Operation**	**Admission**
Concussion and wounds.	No operation.	ICU 2 days + Paediatric Surgical ward 1 day → transfer to primary hospital.
Dislocation of the left sternoclavicular joint.	1. Closed reduction.	Paed. Orthopaedic ward 5 days.
	2. Open reposition and osteosuture.	
Splenic injury.	No operation.	ICU 2 days + Paed. Surgical ward 8 days.
Traumatic right fronto-temporal contusion + base of skull fracture going from the left mastoid into the inner ear with hematotympanon, discontinuity and dislocation of the ossicular chain.	Neurosurgical external ventricular drainage for ICP monitoring and ENT operation of the ear.	Neurosurgical ICU 12 days (ventilator for 10 days) + Paed. Surgical ward 2 days. Rehabilitation.

N = 4.

**Table 3:** Types of injuries, treatment and time at ward in the adult group

**Injury**	**Operation**	**Admission**
Distal humerus fracture.	Open reduction + plate fixation + osteosynthesis.	Orthopaedic ward 4 days.
Subtrochanteric femoral fracture.	Open reposition + fixation nail.	Orthopaedic ward 4 days.
Pneumothorax + multiple rib fractures.	Chest drain placement.	Acute surgical ward 4 days.
Mandibular fracture, superficial oral wound, alveolar process fracture.	Open reduction and osteosynthesis of the mandible + five tooth extractions.	ENT ward 5 days.
Multiple facial fractures and lacerations.	Open reduction, internal fixation and suture of soft tissue injury.	ENT ward 5 days.
Multiple rib fractures. Pneumothorax, contusion of thorax.	Chest drain placement.	ICU 1 day + Acute surgical ward 6 days.
Traumatic hemo-pneumothorax, contusion of thorax, multiple rib fractures.	Chest drain placement.	Acute surgical ward 7 days.
Traumatic liver rupture.	No operation.	Acute surgical ward 7 days.
Sacral fracture.	No operation.	Orthopaedic ward 7 days.

N = 9.

In total, 67/90 CT scans in 31/41 patients were done and 174/183 skeletal x-rays were performed in 98/97 patients.

Operations were performed in 12/11.

In Children there was external stabilization in 24, antibiotics and/or tetanus booster in three, paramedical treatment in six, suture in three and reduction of a finger in one.

In Children twelve operations were performed on 11 patients; one patient had a craniotomy with drainage of an intracranial haemorrhage and placement of an intraventricular drain. Other operations included three open reductions, seven closed reductions, one closure of an intraoral wound and treatment of a dental dislocation.

In Adults there were three chest drain placements, five open reductions of fractures, two closed reductions and one open re-construction of a finger tendon.

Of the total number 65/55 patients received some kind of treatment. There were in total 86/78 treatments (including admission for observation). Apart from operations and admissions, the treatments included stabilization and immobilisation 24/13, paramedical treatment (n = 6/12, suture 3/6, reduction 1/4.

No patient died.

### Costs

The total cost for consultation and treatment was (Children/Adults) 210 000/200 000 Euro, which means approximately 1 400/1 400 euro per accident. The cost for radiological investigation was 24 000/29 000 Euro (CT scans 14 000/18 000 Euro and plain films 10 000/11 000 Euro). Admission and hospital stay 76 000/32 000 Euro. The Eemergency treatment cost 75 000/78 000 euro and operation costs was 36 000/58 000 Euro.

In the Adult group a total of 23 patients (16%) had to be home from work on sick leave after the accident, from 4-135 days and in total, 712 days. Six patients were home on sick leave for one month or more (Table 4).

**Table 4:** Number of patients and reasons for sick leave ≥ 30 days

**Injury**	**Days of sick leave**
Pneumothorax + multiple rib fractures.	30
Calcaneal fracture.	30
Hip distortion.	35
Fracture of the lateral malleolus + injury to the syndesmosis.	56
Distal humeral fracture.	115
Multiple facial fractures and lacerations.	135

N = 6.

### Incidence

There are approximately 4 500 horses in the community of Linköping. Each horse is ridden approximately one hour a day and taken care of for approximately two hours a day. Institutional horses are usually ridden for 2-3 hours a day. This gives an estimated total time of at least 13 500 hours a day of horse handling time, and an accident rate of approximately 0,029/100 000 riding/caretaking hours. The numbers are an estimate achieved from the county administration board of Östergötland and the equestrian’s federation of the county.

### Safety equipment

There were no significant difference between users and non-users of safety equipment.

## Discussion

There was a dominance of females in our study population (98% among Children, 91% among Adults) reflecting the fact that horse riding is more common among females. There was a higher rate of Adults (31%) being injured while dismounted than Children (11%). This is likely due to the fact that adults spend more time, before and after riding, taking care of the horse than children do. These findings are in correlation with previous studies presenting a rate of 15-23% [[8]-[11]].

Comparing injury mechanisms between Children and Adults; 85% of the Children had fall accidents, 7% were kicked, while 65% of the Adults had fall accidents and 16% were kicked. Minor sprains and soft tissue injuries were the most common type of injury in both groups, corresponding to the findings of Kiss et al. [[10]] who stated that contusions to the body were the most common injuries. In both groups the fractures were mainly located in the upper extremity and usually followed falling injuries.

The distribution of injuries in total corresponds to previous studies [[12]]. The most commonly injured sites were the extremities, head and neck. In particular, head and neck injuries were more common among Children (n = 33) than Adults (n = 11), which reflects the fact that children have more falling accidents. With exception of two cases, head injuries were minor which corresponds with previous studies. Head injuries today are less severe than previously, due to improvements in helmet technology and use [[13]].

In a retrospective study by Ghosh et al. [[14]] there were 8 deaths, 40% of patients required ICU and 39% needed surgical intervention. The higher mortality and morbidity rates probably reflects that the study only investigated admitted cases, however even if we only consider our admitted patients, we still find that only 4% of Adults and 8% of Children required ICU treatment, 8% in both groups needed surgical intervention and there were no deaths. We can’t explain this difference from the data given by Gosh et al.

A two year prospective study by Berg et al. [[15]] in another hospital in Östergötland during 1978-80 reported a rate of 29% for admissions between 2-30 days and 33% requiring sick leave ≥30 days. This can be compared with our findings of 5% for admissions ≥3 days, and 4% for those requiring sick leave ≥30 days among Adults. The decreased need for sick leave and admission rate possibly reflects the changes in management of these patients over the years. It might even reflect an increased use of safety equipment. Cuenca et al. [[16]] find an admission rate of 43% which differs from the literature of 5-15% [[9],[17],[18]]. In our study we find an overall admittance of 63 patients (21%).

The rate of treatment in Children and Adults is about the same (44%/39%), showing that the majority of accidents results in minor injuries without need for treatment.

Serious injuries were defined as cases involving hospitalization for ≥3 days (n = 13) and sick leave for ≥1 month (n = 6). We observed 19 (7%) severe. In comparison this is a low figure.

A limitation in this study is that there is no official registry of the number of horses. The incidence rate that we have calculated reflects the fact that our study includes all accidents. When investigating racing sports for instance eventing figures are much higher [[19]].

Horse riding is the eighth most popular sport in Sweden, the number of accidents is less than expected and the severity less than previously stated.

Since the University Hospital in Linköping is the only hospital in the Linköping community (population of 150,000) that takes care of emergency cases, including horse accidents, we assume that the cases of horse related trauma in our study represents the typical horse riding population.

The total cost for medical treatment of horse riding related injury was approximately 400 000 Euro/year. In addition to this, Adults were on sick leave for a total of 712 days.

There was an estimated incidence of horse related injuries of 0.029/100 000 riding/caretaking hours. Further we found that falling from the horse was the most common mechanism of injury. Most injuries were located in the extremities but common also in the head and neck, which is in correlation with the literature.

## Conclusions

Horse riding is a sport with well-known risks [[4],[20]]. Our results are in line with the literature in many instances, however we have not seen the same incidence of serious injuries, and find these to be fairly uncommon. The injuries are mainly minor with small risk of long term morbidity. Over time, regulations and safety equipment seems to have decreased the number of accidents, especially the serious injuries and made horse riding a relatively safe sport to perform.

## Competing interests

The authors declare that they have no competing interests.

## Authors’ contributions

JA: Carried out the collection of data, contact and logistics with all participants and drafted the manuscript. SR: Carried out planning of the entire work, statistics and drafted the manuscript. NH: Collection of data, drafted the manuscript. PD Planning, statistics and drafted the manuscript. All authors read and approved the final manuscript.
